# Distress and Rewards of Nurses with Experience in COVID-19 Wards: A Qualitative Study

**DOI:** 10.12688/f1000research.147675.1

**Published:** 2024-05-31

**Authors:** Asako Matsuura, Shin-ichiro Sasahara, Hirokazu Tachikawa, Keiko Wataya, Masana Ujihara, Yoshitaka Kawashima, Sho Takahashi, Kei Muroi, Shotaro Doki, Daisuke Hori, Tsukasa Takahashi, Ichiyo Matsuzaki

**Affiliations:** 1 Graduate School of Comprehensive Human Sciences, University of Tsukuba, Tsukuba, Ibaraki Prefecture, Japan; 2 Faculty of Health Science, Toho University, Funabashi, Chiba, Japan; 3 Institute of Medicine, University of Tsukuba, Tsukuba, Ibaraki Prefecture, Japan; 4 College of Nursing and Nutrition, Shukutoku University, Chiba, Chiba Prefecture, Japan; 5 School of Arts and Letters, Meiji University, Chiyoda, Tokyo, Japan; 6 International Institute for Integrative Sleep Medicine, University of Tsukuba, Tsukuba, Ibaraki Prefecture, Japan

**Keywords:** COVID-19, Nurse, Distress, Rewards, Dilemma

## Abstract

**Background:**

Amidst the global escalation of COVID-19, nurses have confronted the dual challenge of exposure to infection and the duty to provide patient care, leading to some moral dilemmas. This study aims to explore the psychological burden and dilemmas faced by nurses working in COVID-19 wards, elucidating their professional distress and rewards, and examining their interrelation.

**Methods:**

This qualitative descriptive study employed semi-structured interviews to gather data on the experiences of nurses who worked in COVID-19 wards. The study spanned from January 2022 to March 2023. Qualitative content analysis was applied to analyze interview transcripts.

**Results:**

The study involved 12 participants (8 women and 4 men). Their experience ranged from 4-21 years. The group included 6 staff nurses, 3 head or deputy head nurses, and 3 head nurses. No significant changes were observed in weekly working hours pre- and post-COVID-19. Analysis of the interviews revealed that nurses working in COVID-19 wards experienced conflicts related to the risk of infection at work, role execution, organizational challenges, and interpersonal relationships. Concurrently, they also reported finding rewards in their work and in building connections with others.

**Conclusions:**

This study revealed that nurses experienced distress related to COVID-19-related job challenges, leading to a sense of mistrust towards their organizations. However, working in COVID-19 wards also brought a renewed sense of job fulfillment, particularly through interactions with individuals they had not previously encountered. These experiences are illustrative of the dilemmas faced by healthcare professionals in balancing the distress and rewards inherent in their roles.

## Introduction

The Coronavirus Disease 2019 (COVID-19) pandemic has posed severe challenges in healthcare settings worldwide. Nurses in COVID-19 wards have confronted arduous conditions, balancing the high risk of infection with the responsibility of patient care. Their role extended beyond professional duties to encompass ethical responsibilities, facing a relentless task of managing stringent infection control while simultaneously providing emotional care to patients ( Alloubani et al., 2021). This dual role has placed them in a position of constant striving for professional excellence amid concerns for personal safety and the risk of transmitting the virus to their families, often leading to social isolation both within and outside the hospital.

The high physical and psychological demands of working in COVID-19 wards are well-documented ( Oda et al., 2020; Lai et al., 2020). This burden included moral dilemmas, described as conflicts arising from moral judgments that cannot be acted upon due to constraints, or the uncertainty of what constitutes the right moral decision ( Jameton, 1993). These dilemmas are common in healthcare, where professionals regularly face situations that require choosing between conflicting moral imperatives. Such scenarios, where fulfilling two strong obligations simultaneously is impossible, are notably prevalent ( Fourie, 2015).

During the COVID-19 outbreak, nurses were forced into making difficult decisions regarding resource allocation and setting medical priorities ( Rushton et al., 2022; Greenberg et al., 2020). The challenges of having to make ethically challenging decisions regarding resource distribution, treatment provision, and patient triage had a profound impact on nurse’s mental health ( Lake et al., 2021). Furthermore, the clash between personal ethical beliefs and institutional policies added to the nurses’ stress and mental burden, making the balance between professional ethics and organizational directives a complex issue ( Torkaman et al.,2020). Discrepancies between individual morals and organizational policies have been shown to worsen mental health issues among healthcare workers ( Walton et al., 2020).

The concept of moral injury, which has gained particular attention in the COVID-19 context, refers to the psychological harm that occurs when actions, or lack thereof, violate one’s moral or ethical code ( Greenberg et al., 2020). Such moral injuries have been linked to increased burnout and attrition ( Dalmolin et al., 2014). This moral injury, stemming from moral distress ( Čartolovni et al., 2021), leads to psychological discomfort and negative emotions, which can adversely affect patient care, decrease job satisfaction, and contribute to burnout and resignation ( Deschenes et al., 2020). Therefore, it is crucial to focus on and elucidate the professional distress of nurses working in COVID-19 wards to prevent the deterioration of healthcare workers’ mental health.

Burnout is associated with the intention to leave and the ability to perform job duties ( Dyrbye et al., 2019) and has been identified as a mediator between job satisfaction and the intention to leave ( Ran et al., 2020). The high turnover rate of nurses presents a significant challenge, making it vital to prevent burnout and resignation to maintain healthcare quality. There is a paucity of studies detailing the specific struggles of nurses who worked in COVID-19 wards. Identifying these struggles can help to prevent mitigate the psychological burden and stress on nurses. Furthermore, research on nurse job satisfaction has highlighted the importance of professional relationships among nurses and patient care ( Utriainen and Kyngäs, 2009). Various factors have been reported to influence job satisfaction, including work shifts, leadership, job performance, organizational commitment, effort, and reward styles, and their outcomes, including workplace environment, empowerment, organizational commitment, professional commitment, work stress, and patient satisfaction ( Lu et al., 2019). However, research specifically detailing the job satisfaction and challenges of nurses working in COVID-19 wards is lacking. Therefore, the aim of the current study was to elucidate both the struggles (occupational distress) and satisfactions (sense of achievement) of nurses who worked in COVID-19 wards, as well as their characteristics, and explore how these aspects are interrelated.

## Methods

### Research design

A qualitative descriptive study design using semi-structured interviews was adopted for this study. This study was guided by the Consolidated Criteria for Reporting Qualitative Research (COREQ) ( Tong et al., 2007).

### Participants

The study participants were nursing professionals who have experience of working in a COVID-19 ward. The participants were employed at a large university hospital in the Kanto region of Japan. The hospital restructured general wards to accommodate infectious disease treatment in response to the COVID-19 outbreak. This ward, previously designed for treating infectious diseases, began operating as a COVID-19 ward after the hospital’s decision to admit COVID-19 patients. Pre-pandemic, nurses working in the general wards were requested to serve voluntarily for about two months in this infectious disease ward. Subsequently, after its conversion to a COVID-19 ward, the hospital implemented a three-month rotation system for nursing staff, based on a roster of volunteers who had served in the infectious disease ward before the pandemic. Participant recruitment was conducted by explaining the purpose and methods of the study to facility administrators, both verbally and in writing, and obtaining their consent. Following this, invitations to participate were extended to potential participants using posters and other methods.

### Data collection

The study was conducted from January 2022 to March 2023, with interviews carried out from March 2022 to January 2023. Participants provided demographic information on an interview sheet including gender, years of experience, position, educational background, and weekly working hours before and after the onset of the COVID-19 pandemic. The interviews, conducted semi-structurally, focused on encouraging the nurses to reflect on their experiences working in COVID-19 wards. Interviews were conducted by a trained psychiatrist and occupational physician who are co-researchers in this study.

The content of the interview guide was as follows:

a) Thoughts upon learning about the operation of COVID-19 wards.
b) The extent of consultation with others.
c) Reflections on actions that could have been beneficial at the time.
d) Messages to their past selves.

### Data analysis

The transcription and analysis of the interview data were initially undertaken by the first author, and then conducted in collaboration with two other research team members who are nursing professionals. This was followed by a verification process involving the principal investigator and five additional members holding doctoral degrees. The interview data were analyzed utilizing methods of qualitative content analysis, specifically summary content analysis and exploratory content analysis ( Flick, 2011). Summarizing content analysis involved rephrasing the data, eliminating insignificant or repetitive statements, and bundling similar expressions. Explanatory content analysis clarified ambiguous or contradictory statements by considering the context of their articulation. Transcripts were carefully summarized and analyzed, ensuring contextual integrity, and then abstracted to higher levels of generalization. Similar meanings were grouped into categories and subcategories. The results were repeatedly reviewed by co-researchers to ensure reliability and validity until consensus was reached.

### Ethical considerations

Interviews were conducted in private rooms to ensure privacy, and data were analyzed with personal information excluded. This study was approved by the University of Tsukuba Medical Ethics Committee (Approval No.: 1690; Approval date: December 24, 2021). The study’s purpose and methods were explained both verbally and in writing to the facility administrators, and their consent was obtained before inviting participants. Participants were informed about the voluntary nature of participation, the possibility of withdrawing consent without disadvantage, anonymity, data security, and the publication of results. Consent was documented in writing before conducting the interviews.

## Results

### Overview of study participants

Twelve nurses, 8 women and 4 men, participated in the study ( Table 1). The average interview duration was 20.5 minutes. Age distribution included one in their 20s, four in their 30s, five in their 40s, one in their 50s, and one in their 60s. Years of experience ranged from four to over 20 years. The group comprised six staff nurses, three deputy head nurses, and three head nurses. The average weekly working hours were 44.2 before COVID-19 and 43.3 after.

**Table 1.** Overview of participant demographics ( *N* = 12).

Attribute	Detail	*N*
Participants	Males	4
	Females	8
Age group	20s	1
	30s	4
	40s	5
	50s	1
	60s	1
Years of experience	4-10 years	4
	11-20 years	4
	Over 21 years	4
Position (at the time)	Staff	6
	Deputy Head Nurse	3
	Head Nurse	3
Educational Background	Vocational School (3-year program)	4
	Junior College (3-year program)	2
	University (4-year program)	6
Work Hours/Week	Pre-COVID-19	44.2 hours/week
	Post-COVID-19	43.3 hours/week

### Distress experienced by nurses working in COVID-19 wards

Analysis of interview content yielded 192 codes, 6 categories and 18 subcategories. Table 2 shows the four categories and 14 subcategories that were identified to describe the distress experienced by nurses working in COVID-19 wards. The four categories were: Risk of infection-related conflicts, Challenges in fulfilling job roles, Organizational conflicts, and Interpersonal conflicts.

**Table 2.** Distress experienced by nurses in COVID-19 wards.

Category	Subcategory	*N* (%)
Risk of Infection-Related Conflicts	Fear of contracting COVID-19	5 (2.6)
	Stress from not discussing ward duties,	4 (2.0)
	Concern for infected or exposed staff	3 (1.5)
	Anxiety about family infection	1 (0.5)
	Struggles with family and societal relations	9 (4.6)
Challenges in Fulfilling Job Roles	Frustration in role fulfillment	11 (5.6)
	The thought of why I have to do it	17 (8.7)
	Stress from unfamiliar tasks	20 (10.2)
	Anxiety about not knowing what the future holds	11 (5.6)
	Criticism from other departments	4 (2.0)
	Inadequacy in managerial roles	16 (8.2)
Organizational Conflicts	Frustration over inadequacies of the work system	11 (5.6)
	Desire for better responsiveness from upper management	17 (8.7)
Interpersonal Conflicts	Difficulty in establishing new relationships	25 (12.8)

With regards to the first category, Risk of infection-related conflicts, participants expressed fears related to COVID-19, with statements such as, “I was scared because I didn’t know where I might get infected,” “Before the vaccine rollout, there was a lot of fear,” and “During outbreaks, I didn’t want to work in the COVID-19 ward.” These statements reflect their fear of contracting the virus. Amidst these fears, one participant mentioned concealing their assignment to the COVID-19 ward due to concern about others’ perceptions, and was shocked upon overhearing conversations filled with prejudice against COVID-19. This led to stress about not being able to disclose their work in the COVID-19 ward openly.

Additionally, working in the COVID-19 ward triggered fears of becoming infected or a carrier, leading to self-critical thoughts like, “Even as a nurse, I worry about getting infected.” One mentioned how hearing of a nurse contracting the virus at a social gathering, despite hospital advisories against such gatherings, sparked negative feelings. There were also concerns for colleagues who became infected or were close contacts, reflecting a deep empathy for fellow staff members.

Working in a COVID-19 ward also brought anxiety about potentially infecting family members, compounded by societal prejudices. This resulted in worries about family and social relationships, with instances of biased neighbors or relatives making hurtful comments. The burden this placed on family members was another source of concern for the participant.

Regarding the second category, Challenges in fulfilling job roles, nurses expressed frustration related to their roles, with sentiments like, “I don’t want to stress the younger staff,” and “We need to understand it’s part of our ward duties.” Despite these feelings, they faced situations where temporary transfers became permanent, and expressed reluctance, saying, “I’m not doing this because I want to,” “If I could avoid it, that would be best,” and “Being single and living alone, it seems inevitable I’ll be sent.” These statements revealed a sense of obligation and questioning of why they must shoulder these responsibilities. There was also a sense of resignation, with thoughts like, “I can do this [my duties] because it won’t last forever,” and “Time will solve this,” indicating hope that time might ease the struggle with unfamiliar tasks. However, they still experienced stress due to these new duties. Amidst this vague anxiety, nurses wondered, “Where will I be in a few weeks?” and felt uneasy about being transferred to unknown wards without knowing what lies ahead, thinking, “Will I keep moving between wards forever?” and “There was never a moment of reassurance,” reflecting their anxiety about the uncertain future.

Despite these various stresses and anxieties, nurses noted differences in cooperation between wards and faced criticism from other departments, with remarks like, “You must be having an easy time now that the number of infections has decreased.” Those in managerial positions felt they were doing their best but often found themselves uncertain about what to do, feeling blamed and without the capacity to respond, leading to a sense of inadequacy in fulfilling their roles as nursing managers.

The category of Organizational conflicts highlighted the frustrations nurses experienced due to operational inefficiencies in the COVID-19 ward, such as “feeling helpless because preparations were inadequate” and “a negative atmosphere due to many uncertainties.” These issues contributed to a sense of difficulty in performing their duties. Nurses expressed a desire to share these challenges with the organization, feeling that “the extent of the difficulties wasn’t fully understood by everyone” and “there was a lack of effective communication with the higher-ups.” This led to a growing sentiment of wanting more support and understanding from the upper management. Furthermore, the experience led to a negative perception of the organization, with nurses realizing that “it’s an organization that oppresses and utilizes a top-down approach.” There was a collective sense of anger towards the upper management, indicating a deepening mistrust and dissatisfaction among the staff.

Regarding the final category, the necessity of building new relationships arose for those transferred to the COVID-19 ward. There was a sense of a weakened team spirit, as described by phrases like, “It felt like there was little camaraderie.” Nurses and doctors reported feeling unable to seek advice, leading to situations where “supervisors felt isolated.” This highlighted a scenario where establishing relationships with colleagues and the organization proved challenging. Amidst these struggles, a manager mentioned, “While it was good to hear everyone’s stories, it was also difficult.” This statement reflects the complexity of fostering relationships within the team. The hindrance in these efforts was attributed to poor relationships, as expressed in the comment, “The bad relationships became a constraint.”

### Job rewards gained from working in COVID-19 wards

Two categories, Job rewards and Forming new human connections, were identified as more positive experiences gained from working in the COVID-19 wards ( Table 3).

**Table 3.** Job rewards gained from working in COVID-19 wards.

Category	Subcategory	*N* (%)
Job rewards	Sense of mission and responsibility	8 (4.1)
	Insights gained from experience	22 (11.2)
Formation of New Human Connections	Collaboration among staff	8 (4.1)
	Unity as an organization	4 (2.0)

In the first category, Job rewards, the nurses described feeling a sense of mission and responsibility, gaining rewards from treating patients, and finding social significance in accepting them. Some viewed their experience positively as an opportunity for personal growth, gaining new insights and confidence through their work.

The second positive aspect was the category of Formation of new human connections in which participants describe how they found value in forming connections with staff from other departments and experienced a sense of unity within the ward, leading to cohesive teamwork.

## Discussion

This study revealed that nurses with experience of working in COVID-19 wards faced a blend of distress and fulfillment in their duties. Consistent with prior research ( Awano et al., 2020), these nurses grappled with fears directly related to COVID-19, such as the fear of infection. This aligns with the broader body of research focusing on the stress and psychological impact faced by healthcare workers during the COVID-19 pandemic ( Arias et al., 2023). Our study underscores how nurses working in infectious disease wards confronted unique dilemmas and distress. Additionally, the pandemic’s impact extended beyond the individual nurse to their families, placing nurses in a difficult position of balancing personal and family safety with their professional obligations ( Catania et al., 2021). In such scenarios, a safe and secure organizational environment is crucial for enabling nurses to fulfill their duties. However, previous reports have indicated a sense of distrust towards organizations and conflicts in interpersonal relationships among nurses during the COVID-19 pandemic ( Soto et al., 2020). Our study also highlighted these aspects, and it is likely that accumulation of such conflicts over time will lead to distress and potentially to burnout. Lack of communication and support within organizations might have exacerbated nurses’ stress, forming the root cause of their distress and dilemmas. Organizational support is crucial in maintaining nurses’ mental health ( Labrague, 2021), with managerial presence, information provision, and attentiveness to subordinates’ opinions reducing nurses’ anxiety and facilitating mutual understanding under challenging work conditions (Matilda S. et al., 2022). This suggests the importance of creating a safe and secure environment for nurses. Future strategies should focus on how to support nurses when they face such conflicts.

Importantly, this study also revealed certain positive aspects, such as job rewards, a sense of mission, and the forging of new relationships and connections, despite the challenges faced in COVID-19 wards. This could be related to the joy and rewards healthcare workers derive from helping others and reconnection with a sense of vocation. Reports suggest that contributions to patient care and a sense of accomplishment positively impacted healthcare workers’ mental health during the COVID-19 pandemic ( Yamada et al., 2022; Trumello et al., 2020). These findings imply that working in COVID-19 wards and responding to patients’ needs can cultivate a sense of professionalism and fulfillment. Onodera (2022) noted that in wards temporarily formed with members from different departments, the ability to collaborate is crucial for enhancing caregiving capabilities. Collaboration involves building relationships with team members, identifying and solving workplace problems, understanding the abilities of members from different specialties, and appropriately delegating tasks. The findings of our study suggests that nurses from various wards working together shared diverse expertise, leading to the formation of staff collaboration and organizational unity. Working while forming new human connections may have also enhanced nurses’ caregiving capabilities. The novel environment of the COVID-19 wards potentially enhanced the professional capabilities of each individual, as they were compelled to utilize their skills to the fullest. A key factor in drawing out these abilities may have been the necessity to form relationships with colleagues they had never worked with before.

Nurses, as healthcare professionals, faced the need to respond to the unfamiliar challenge of COVID-19, carrying with them feelings of anxiety and distress. In this context, they not only experienced personal growth triggered by COVID-19 but also expanded their professional connections. This situation revealed a dilemma between the struggles and the rewarding nature of their work, shedding light on the evolving aspects of their professional dilemmas.

### Limitations and future prospects of this study

This study is limited by its focus on nursing professionals from a single facility, rendering the results non-generalizable. To construct a comprehensive theory, further data collection and analysis involving other institutions and a larger sample size are necessary. Additionally, capturing temporal changes was challenging, necessitating ongoing monitoring of the situation.

Based on the insights gained from this research, it’s essential to conduct quantitative studies to explore the content of nurses’ conflicts, especially during times of societal emergencies. This will contribute to the development of effective strategies and interventions to support nurses during such times. Moreover, a more detailed examination of the types of psychological support that can be offered to nurses is required to effectively address their specific needs and challenges. Nurses, as professionals, faced the necessity to respond to COVID-19, an unknown infectious disease, which brought with it severe feelings of anxiety and distress. Amidst these challenges, the nurses in our study also experienced personal growth and an expansion of connections with others, triggered by COVID-19. This has revealed how they, as professionals, are grappling with the dilemma of experiencing both distress and fulfillment, and their perspectives on how to perceive and manage this dilemma moving forward. Further investigations into both the negative and positive aspects brought by the pandemic can help us to understand how to support nurses in their vital caregiving work.

## Conclusion

This study sought to elucidate the distress and dilemmas experienced by Japanese nurses working in COVID-19 wards. It revealed that nurses faced significant challenges, including conflicts related to the risk of infection, challenges in fulfilling job roles, organizational conflicts, and difficulties within interpersonal relationships. Despite these challenges, nurses also found positive aspects such as job rewards and opportunities to form new human connections throughout their experiences working on COVID-19 wards. These findings indicate that while nurses bore the distress and burden related to COVID-19, leading to mistrust towards their organizations, they simultaneously discovered new aspects of job satisfaction and the value of engaging with people they had not previously encountered. Our findings illustrate the dilemmas faced by professionals in handling the distress inherent in their roles and balancing that with their sense of professional duty and vocational rewards. The insights gained from our investigation into the experiences of nurses working on COVID-19 wards at the height of the deadly pandemic can help healthcare providers to devise effective strategies and interventions to support nurses in their important work.

## Ethics and consent

This study was approved by the University of Tsukuba Medical Ethics Committee (Approval No.: 1690) Approval date: December 24, 2021. The study’s purpose and methods were explained both verbally and in writing to the facility administrators, and their consent was obtained before inviting participants. Participants were informed about the voluntary nature of participation, the possibility of withdrawing consent without disadvantage, anonymity, data security, and the publication of results. Consent was documented in writing before conducting the interviews.

## Authors’ contributions

Asako Matsuura contributed to the research design, data collection, analysis and interpretation, and drafting of the manuscript. Shin-ichiro Sasahara was involved in conceptualizing the study, research design, recruiting participants, and contributed to data collection, analysis, and interpretation. Hirokazu Tachikawa contributed to the conception of the study, interpretation of the data. Yoshitaka Kawashima and Sho Takahashi contributed to interpretation. Shotaro Doki, Daisuke Hori, and Tsukasa Takahashi assisted in recruiting participants and contributed to data analysis and interpretation. Masana Ujihara and Keiko Wataya contributed to the analysis and interpretation of research data and critically revised the content of the manuscript. Kei Muroi contributed to the critical revision and English proofreading of the manuscript. All authors discussed the results critically and approved the final version of the manuscript.

## Data Availability

Parts of the data used in this study are available for public access at Matsuura (2024). However, from the perspective of personal information protection, specific details of certain datasets remain confidential. This includes data deemed inappropriate for public release because it contains information that could identify individuals. For detailed inquiries about the use of data in this research, or if you wish to request limited access to the data, please contact the principal investigator directly. Zenodo: Distress and rewards of nurses with experience in COVID-19 wards. https://zenodo.org/doi/10.5281/zenodo.10616718 ( Matsuura, 2024). This project contains the following data:
- Interview Data.docx - interview guide.docx -
Table 1.xlsx -
Table 2.xlsx -
Table 2_code.xlsx -
Table 3. xlsx -
Table 3_code.xlsx Data are available under the terms of the Creative Commons Attribution 4.0 International license (CC-BY 4.0).
