# The Dilemmas, Distresses, and Rewards Experienced by Nurses Working in COVID-19 Wards: A Qualitative Study

**DOI:** 10.12688/f1000research.147675.2

**Published:** 2025-04-22

**Authors:** Asako Matsuura, Shin-ichiro Sasahara, Hirokazu Tachikawa, Keiko Wataya, Masana Ujihara, Yoshitaka Kawashima, Sho Takahashi, Kei Muroi, Shotaro Doki, Daisuke Hori, Tsukasa Takahashi, Ichiyo Matsuzaki

**Affiliations:** 1 Graduate School of Comprehensive Human Sciences, University of Tsukuba, Tsukuba, Ibaraki Prefecture, Japan; 2 Faculty of Health Science, Toho University, Funabashi, Chiba, Japan; 3 Institute of Medicine, University of Tsukuba, Tsukuba, Ibaraki Prefecture, Japan; 4 College of Nursing and Nutrition, Shukutoku University, Chiba, Chiba Prefecture, Japan; 5 School of Arts and Letters, Meiji University, Chiyoda, Tokyo, Japan; 6 International Institute for Integrative Sleep Medicine, University of Tsukuba, Tsukuba, Ibaraki Prefecture, Japan

**Keywords:** COVID-19, Nurse, Distress, Rewards, Dilemma

## Abstract

**Background:**

Amidst the global escalation of COVID-19, nurses have confronted the dual challenge of exposure to infection and the duty to provide patient care, leading to some moral dilemmas. This study aims to investigate the dilemmas, occupational and psychological distresses and professional rewards experienced by nurses working in COVID-19 wards and explore their nature and interrelation.

**Methods:**

This qualitative descriptive study employed semi-structured interviews to gather data on the experiences of nurses who worked in COVID-19 wards. The study spanned from January 2022 to March 2023. Qualitative content analysis was applied to analyze interview transcripts.

**Results:**

The study involved 12 participants (8 women and 4 men). Their experience ranged from four-21 years. The group included six staff nurses, three deputy head nurses, and three head nurses, totaling six head nurses in managerial positions. No significant changes were observed in weekly working hours pre- and post-COVID-19. Analysis of the interviews revealed that nurses working in COVID-19 wards experienced conflicts related to the risk of infection at work, role execution, organizational challenges, and interpersonal relationships. Concurrently, they also reported finding rewards in their work and in building connections with others.

**Conclusions:**

This study revealed that nurses experienced distress related to COVID-19-related job challenges, leading to a sense of mistrust towards their organizations. However, working in COVID-19 wards also brought a renewed sense of job fulfillment, particularly through interactions with individuals they had not previously encountered. These experiences are illustrative of the dilemmas faced by healthcare professionals in balancing the distress and rewards inherent in their roles.

## Introduction

The Coronavirus Disease 2019 (COVID-19) pandemic posed unprecedented challenges in healthcare systems globally. Nurses in COVID-19 wards faced demanding situations, striving to balance infection risks with patient care responsibilities. Their role extended beyond professional duties to encompass ethical responsibilities, facing a relentless task of managing stringent infection control while simultaneously providing emotional care to patients ( Alloubani et al., 2021). This dual role placed them in a position of constant striving for professional excellence amidst concerns for personal safety and the risk of transmitting the virus to their families, often leading to social isolation both within and outside the hospital ( Asa et al., 2022).

The systematic reviews and meta-analyses by Varghese A (2021) and García-Vivar C (2023) comprehensively examined the psychological impact of the COVID-19 pandemic on nurses, revealing that nurses experienced significantly higher levels of anxiety, depression, and burnout during the pandemic ( Varghese A et al., 2021; García-Vivar C et al., 2023). These reviews identified infection risk, excessive workload, shortage of personal protective equipment, and ethical dilemmas as the main stressors (Chigwedere et al., 2021). While quantitative research has demonstrates the prevalence of psychological distress, it has also been noted that qualitative investigations remains essential for a deeper understanding of nurses’ subjective experiences, emotions, and cognitive processes in overcoming these challenging situations ( Wong KW et al., 2024).

During the COVID-19 outbreak, nurses were forced into making difficult decisions regarding resource allocation and setting medical priorities ( Rushton et al., 2022; Greenberg et al., 2020). The challenges of having to make ethically difficult challenging decisions regarding resource distribution, treatment provision, and patient triage had a profound impact on nurse’s mental health ( Lake et al., 2021). Furthermore, the clash between personal ethical beliefs and institutional policies added to the nurses’ stress and mental burden, making the balance between professional ethics and organizational directives a complex issue ( Torkaman et al.,2020). Such discrepancies between individual morals and organizational policies have been shown to worsen mental health issues among healthcare workers ( Walton et al., 2020).

The concept of moral injury, which has gained particular attention in the COVID-19 context, refers to the psychological harm that occurs when actions, or lack thereof, violate one’s moral or ethical code ( Greenberg et al., 2020). Such moral injuries have been linked to increased burnout and attrition ( Dalmolin et al., 2014). This moral injury, stemming from moral distress ( Čartolovni et al., 2021), leads to psychological discomfort and negative emotions, which can adversely affect patient care, decrease job satisfaction, and contribute to burnout and resignation ( Deschenes et al., 2020). Therefore, it is crucial to focus on and clarify the professional distress of nurses working in COVID-19 wards to help prevent the deterioration of healthcare workers’ mental health when they face such crises.

Burnout is associated with the intention to leave and the ability to perform job duties ( Dyrbye et al., 2019) and has been identified as a mediator between job satisfaction and the intention to leave ( Ran et al., 2020). The high turnover rate of nurses presents a significant challenge, making it vital to prevent burnout and resignation in order to maintain healthcare quality. There is a paucity of studies detailing the specific struggles of nurses who worked in COVID-19 wards. Identifying these struggles can help to prevent mitigate the psychological burden and stress on nurses. Furthermore, research on nurse job satisfaction has highlighted the importance of professional relationships among nurses and patient care ( Utriainen and Kyngäs, 2009). Various factors have been reported to influence job satisfaction, including work shifts, leadership, job performance, organizational commitment, effort, and reward styles, and their outcomes, including workplace environment, empowerment, organizational commitment, professional commitment, work stress, and patient satisfaction ( Lu et al., 2019). However, research specifically detailing the job satisfaction and challenges of nurses working in COVID-19 wards is lacking. Therefore, the aim of this study was to investigate the dilemmas, occupational and psychological distresses, and professional rewards (sense of achievement) of nurses who worked in COVID-19 wards, and explore their nature and how these aspects are interrelated, ultimately shedding light on the professional dilemmas they encounter.

## Methods

### Research design

This study employed a qualitative descriptive study design utilizing semi-structured interviews to deeply explore the experiences of nurses in COVID-19 wards. To enhance the rigor of qualitative reporting, the study adhered to the Consolidated Criteria for Reporting Qualitative Research (COREQ) ( Tong et al., 2007).

### Participants

The study participants were nursing professionals who had experience of working in a COVID-19 ward. The participants were employed at a large university hospital in the Kanto region of Japan. The hospital had restructured general wards to accommodate infectious disease treatment in response to the COVID-19 outbreak. This ward, previously designed for treating infectious diseases, had begun operating as a COVID-19 ward after the hospital’s decision to admit COVID-19 patients. Pre-pandemic, nurses working in the general wards were requested to serve voluntarily for about two months in this infectious disease ward. Subsequently, after its conversion to a COVID-19 ward, the hospital implemented a three-month rotation system for nursing staff, based on a roster of volunteers who had served in the infectious disease ward before the pandemic. Participant recruitment was conducted by explaining the purpose and methods of the study to facility administrators, both verbally and in writing, and obtaining their consent. Following this, invitations to participate were extended to potential participants using posters and other methods.

### Sampling technique

To recruit participants, a purposive sampling strategy was employed, specifically a criterion-based convenience sampling approach. Given that the study population was narrowly defined to nurses with experience in COVID-19 wards at a single university hospital in the Kanto region of Japan, purposive sampling was deemed optimal to ensure participants possessed the necessary experience relevant to our research questions.

The inclusion criteria were: (1) being a registered nurse; (2) having direct work experience in the COVID-19 ward at the participating university hospital during the study period (January 2022 to March 2023); and (3) being willingness to participate in a semi-structured interview.

Recruitment was conducted in several stages. First, the purpose and methods of the study were explained both verbally and in writing to facility administrators, and their consent was obtained. Seconds, as the hospital’s three-month rotation system for nurses volunteering to work in COVID-19 wards was used to identify potential participants who had served or were serving in COVID-19 wards. Third, posters were displayed in nurses’ lounges and other relevant areas within the hospital to disseminate study information and invite participation. Potential participants were also informed about the study by facility administrators. Fourth, prior to participation, all nurses who expressed interest were provided with detailed information about the study, including its purpose, procedures, voluntary nature of participation, and assurance of confidentiality. Written informed consent was obtained from all participants before the commencement of interviews.

A sample size of 12 participants was determined to be appropriate for this qualitative descriptive study, which aimed to explore experiences and perspectives in depth.

### Questionnaire development

A semi-structured interview guide was developed to ensure focused and comprehensive data collection. The interview guide questions were developed through a multi-stage process. First the study’s aims were reconfirmed, focusing on distress, rewards, and dilemmas experienced by nurses in COVID-19 wards. Subsequently, a review of relevant literature was conducted, including qualitative studies on nurses’ experiences during times of health crises. This review helped identify key themes and areas to explore in the interviews. Next, based on the literature review and research aims, the research team drafted an initial set of open-ended questions. The team, comprising nursing professionals, qualitative research experts, and a psychiatrist, engaged in iterative discussions to refine these questions. These discussions focused on ensuring the questions’ relevance, comprehensiveness, clarity, and appropriateness for eliciting useful data from participants. The questions were primarily open-ended to encourage participants to freely share their experiences and perspectives, aiming to capture a wide range of experiences related to distress and rewards. The final interview guide was structured around four main categories designed to comprehensively explore the nurses’ experiences:

1. Initial Reactions to COVID-19 Ward Assignment: To understand their thoughts and feelings upon learning about their assignment to a COVID-19 ward.
2. Consultation with Others: To explore the extent to which nurses sought advice or support from colleagues or others.
3. Reflections on Beneficial Actions: To encourage nurses to reflect on actions or behaviors that they found helpful or beneficial during their experiences.
4. Messages to Past Selves: To provide an opportunity for nurses to offer advice and reflect on their journey through the pandemic, offering a message to their past selves.

The preliminary interview guide was pilot-tested with two nurses with COVID-19 ward experience (but not included in the main study). Feedback from these pilot interviews led to revisions in question wording, flow, and overall appropriateness. These pilot interviews helped improve the clarity and flow of the questions, ensuring they were easily understood and effectively elicited the intended information. The research team confirmed the appropriateness and sensitivity of the interview guide based on the pilot testing.

### Data collection

The study was conducted from January 2022 to March 2023. Participant recruitment and interview guide development began following ethical approval for the study in January 2022. The interviews, conducted semi-structurally, were conducted by a trained psychiatrist and occupational physician who were co-researchers in this study. Participants also provided demographic information on their interview sheet including gender, years of experience, position, educational background, and weekly working hours before and after the onset of the COVID-19 pandemic.

### Data analysis

The transcription and analysis of the interview data were initially undertaken by the first author, and then conducted in collaboration with two other research team members who were nursing professionals. This was followed by a verification process involving the principal investigator and five additional members holding doctoral degrees. The interview data were analyzed utilizing methods of qualitative content analysis, specifically summary content analysis and exploratory content analysis ( Flick, 2011). Summarizing content analysis involved rephrasing the data, eliminating insignificant or repetitive statements, and bundling similar expressions. Explanatory content analysis clarified ambiguous or contradictory statements by considering the context of their articulation. Transcripts were carefully summarized and analyzed, ensuring contextual integrity, and then abstracted to higher levels of generalization. Similar meanings were grouped into categories and subcategories. Data saturation was considered to have been reached when no new themes or subcategories emerged from the interview data, and existing categories were well-elaborated and richly described ( Saunders et al., 2018). To ensure data saturation, we employed an iterative process of data collection and analysis. After each interview, the transcripts were analyzed, and subsequent interviews were conducted with attention to emerging themes. Data saturation was determined through ongoing discussions among the research team members, including nursing professionals and experienced qualitative researchers, after reviewing the transcripts and codes up to the 10th interview. While we continued to conduct two more interviews (total 12) to confirm saturation, no new significant themes or subcategories were identified, reinforcing our conclusion that data saturation had been achieved by the 10th interview.

The results were repeatedly reviewed by co-researchers to ensure reliability and validity until consensus was reached.

### Ethical considerations

Interviews were conducted in private rooms to ensure privacy, and data were analyzed with personal information excluded. This study was approved by the University of Tsukuba Medical Ethics Committee (Approval No.: 1690; Approval date: December 24, 2021). The study’s purpose and methods were explained both verbally and in writing to the facility administrators, and their consent was obtained before inviting participants. Participants were informed about the voluntary nature of participation, the possibility of withdrawing consent without disadvantage, anonymity, data security, and the publication of results. Consent was documented in writing before conducting the interviews.

## Results

### Overview of study participants

The study involved 12 participants (8 women and 4 men) with a wide range of experience, from four to 21 years ( Table 1). The participants included six staff nurses, three deputy head nurses, and three head nurses. Weekly working hours remained largely consistent before and after the onset of the COVID-19 pandemic. Qualitative analysis of the interviews identified two overarching categories describing the nurses’ experiences in COVID-19 wards: (1) Distress Experienced by Nurses Working in COVID-19 Wards and (2) Job Rewards Gained from Working in COVID-19 Wards.

**Table 1.** Overview of participant demographics ( *N* = 12).

Attribute	Detail	*N*
Participants	Males	4
	Females	8
Age group	20s	1
	30s	4
	40s	5
	50s	1
	60s	1
Years of experience	4-10 years	4
	11-20 years	4
	Over 21 years	4
Position (at the time)	Staff	6
	Deputy Head Nurse	3
	Head Nurse	3
Educational Background	Vocational School (3-year program)	4
	Junior College (3-year program)	2
	University (4-year program)	6
Work Hours/Week	Pre-COVID-19	44.2 hours/week
	Post-COVID-19	43.3 hours/week

### Distress experienced by nurses working in COVID-19 wards

This overarching category comprised four main themes: (1) Risk of Infection-Related Conflicts, (2) Challenges in Fulfilling Job Roles, (3) Organizational Conflicts, and (4) Interpersonal Conflicts. These four main themes were further divided into fourteen subcategories, which are summarized in Table 2, together with example statements from the interview transcripts to illustrate each subcategory. These themes represent the diverse sources of distress experienced by nurses during the COVID-19 pandemic.

**Table 2.** Distress experienced by nurses in COVID-19 wards.

Category	Subcategory	N(%)	Example statement
Risk of Infection-Related Conflicts	Fear of contracting COVID-19	5(2.6)	I was afraid because I didn’t know where I might get infected.
			Before the vaccine rollout, there was a lot of fear.
	Stress from not discussing ward duties,	4(2.0)	I kept my assignment to the COVID-19 ward secret.
			There was a stark disconnect between the world inside the hospital and the world outside.
			I felt like people treated me as if I were contagious.
	Concern for infected or exposed staff	3(1.5)	People questioned what I, as a nurse, was doing to prevent infection.
			It felt pointless to be told to quarantine without proper infection control being enforced.
			Getting infected after eating together created a lot of negativity.
	Anxiety about family infection	1(0.5)	I was constantly worried about infecting my children.
	Struggles with family and societal relations	9(4.6)	We experienced prejudice from those around us in society.
			My family was worried when I was assigned to the COVID-19 ward.
			I was worried I was placing a burden on my family.
			My family understood the importance of my work.
Challenges in Fulfilling Job Roles	Frustration in role fulfillment	11(5.6)	I had to come to terms with my responsibilities as part of the Infection Control Unit.
			I didn’t want to put extra stress on the younger staff.
	The thought of why I have to do it	17(8.7)	A supposedly temporary transfer became a permanent one.
			I'm not doing this because I want to.
			If I could avoid this situation, I would.
			Since I’m single and live alone, it felt inevitable that I’d be sent.
	Stress from unfamiliar tasks	20(10.2)	We were rushed to meet deadlines, so we just did it.
			I hoped that time would eventually make things better.
	Anxiety about not knowing what the future holds	11(5.6)	I was anxious about being sent to a ward where I didn’t know anyone or what to expect.
			Will I just keep getting moved from ward to ward forever?
			I never had a moment where I felt truly at ease.
			I kept wondering where I’d be working in a few weeks.
	Criticism from other departments	4(2.0)	People assumed things must be easier now that case numbers are down.
			I sensed a lack of unity and coordination between wards.
	Inadequacy in managerial roles	16(8.2)	I really didn’t know what I was supposed to do.
			I didn’t even have time to argue with anyone.
Organizational Conflicts	Frustration over inadequacies of the work system	11(5.6)	It was frustrating not being prepared for this.
			There was so much uncertainty, and the atmosphere was tense.
	Desire for better responsiveness from upper management	17(8.7)	I wished we could talk more and support each other.
			Everyone shared a sense of anger toward upper management.
			I just wanted to stay as uninvolved as possible.
			I was reminded again how this organization relies on top-down control.
			We all felt that our struggles weren’t being understood at all.
Interpersonal Conflicts	Difficulty in establishing new relationships	25(12.8)	I didn’t feel like I was part of a team.
			They wouldn’t even let me talk things through with them.
			Hearing everyone’s experiences was comforting, but also painful.
			Our poor relationships held us back.
			My supervisor ended up completely isolated.

The first theme, Risk of Infection-Related Conflicts: encompassed anxieties and conflicts stemming directly from the risk of infection. A significant subcategory was “Fear of contracting COVID-19,” with nurses expressing statements like, “I was afraid because I didn’t know where I might get infected,” revealing a pervasive fear of personal infection. This fear was often compounded by “Anxiety about family infection,” and “Struggles with family and societal relations.” Nine participants reported difficulties in this area. One nurse described encountering societal prejudice, stating “We experienced prejudice from those around us in society.” They concealed their COVID-19 ward assignment due to concern about negative perceptions, demonstrating the social burden nurses carried. Additionally, “Stress from not discussing ward duties” reflected in comments like “There was a stark disconnect between the world inside the hospital and the world outside,” and “Concern for infected or exposed staff” illustrated by thoughts such as “People questioned what I, as a nurse, was doing to prevent infection,” contributed to this theme, indicating anxieties about managing infection risks for themselves and their colleagues.

Challenges in Fulfilling Job Roles, the second theme, described the distress arising from the difficulties in performing their professional duties within the demanding COVID-19 ward environment. “Stress from unfamiliar tasks” was a major subcategory, with 20 participants reporting anxiety related to new responsibilities. Nurses expressed feeling unprepared and overwhelmed by tasks outside their usual scope of practice, sometimes feeling “We were rushed to meet deadlines, so we just did it.” This was linked to “Frustration in role fulfillment,” as nurses struggled to meet expectations and maintain their professional standards in the face of these new challenges. Sentiments such as, “I didn’t want to put extra stress on the younger staff,” and the reflection “I had to come to terms with my responsibilities as part of the Infection Control Unit.” “We were rushed to meet deadlines, so we just did it,” revealed a sense of obligation mixed with reluctance regarding their constantly changing roles. The “Thought of why I have to do it” and “Anxiety about not knowing what the future holds” illustrated by concerns like “I was anxious about being sent to a ward where I didn’t know anyone or what to expect,” and “Will I just keep getting moved from ward to ward forever?”, further illustrated their uncertainty and emotional burden. Moreover, “Criticism from other departments” added to their distress, as nurses felt misunderstood and undervalued by colleagues outside the COVID-19 wards, with comments like, “People assumed things must be easier now that case numbers are down.” Finally, “Inadequacy in managerial roles” was reported by managerial staff who felt uncertain and blamed, leading to feelings of inadequacy in their leadership capacity.

Organizational Conflicts, captured the distress stemming from systemic and organizational issues. “Frustration over inadequacies of the work system” was a central subcategory, indicating nurses’ feelings of helplessness due to inefficient systems and inadequate resources. One nurse’s statement about “There was so much uncertainty, and the atmosphere was tense” exemplifies this. The negative atmosphere caused by the many uncertainties within the organization also contributed to their distress. A strong “Desire for better responsiveness from upper management” emerged, with nurses feeling a lack of support and understanding from leadership. They expressed a need for more effective communication and recognition of the challenges they were facing, feeling that “Everyone shared a sense of anger toward upper management.” and “We all felt that our struggles weren’t being understood at all.” This lack of support created a sense of mistrust and dissatisfaction, leading to a perception of the organization as one “I was reminded again how this organization relies on top-down control.”

Interpersonal Conflicts, the final theme to emerge, describes the distress associated with difficulties in forming and maintaining relationships in the rapidly changing COVID ward environment. The subcategory, “Difficulty in establishing new relationships,” in indicated the challenges of building cohesive teams quickly with constantly changing staff. The feeling of weakened team spirit, described as “It felt like there was little camaraderie,” through statements like “I didn’t feel like I was part of a team,” and the sense that “My supervisor ended up completely isolated” illustrate the interpersonal struggles nurses faced in forming supportive professional connections. One manager’s comment, “Hearing everyone’s experiences was comforting, but also painful.” reflects the complexity of sustaining relationships amidst the high-stress environment. One comment, “Our poor relationships held us back” hindered effective teamwork and mutual support.

### Job rewards gained from working in COVID-19 wards

Counterbalancing the distress, nurses also described positive aspects and rewards derived from their experiences, categorized into two main themes (1) Job Satisfaction and (2) Formation of New Human Connections – and four subcategories, as shown in Table 3 including example statements from the interview transcripts to illustrate each subcategory.

**Table 3.** Job satisfaction gained from working in COVID-19 wards.

Category	Subcategory	Example statement
Job satisfaction	Sense of mission and responsibility	Even though patients were struggling, my role as a nurse didn’t change.
		As hospital admissions increased, we told ourselves, “Let’s get through this together!”—and then, suddenly, it was time to move on.
	Insights gained from experience	It turned out to be an incredible experience.
		It’s not that we wanted to do it, but we saw it as a valuable experience.
		There were many things I didn’t fully understand at the time.
		Being assigned to the COVID-19 ward changed the way staff felt empowered.
		It gave me a chance to build confidence.
		I was genuinely interested in working on the front lines of the COVID-19 response.
		Being there firsthand helped me truly understand the reality of infection.
Formation of New Human Connections	Collaboration among staff	I was able to connect with colleagues from other departments.
		I heard many different perspectives from staff across various wards.
		Communication among staff members improved.
	Unity as an organization	The wards became more unified.
		We truly came together as one team.
		Discussions about how to manage the wards brought people together.

The first theme, Job Rewards, encompassed the intrinsic and extrinsic rewards experienced through their work. A core subcategory was “Sense of mission and responsibility,” with nurses expressing a heightened feeling of purpose and contribution during the pandemic, reflected in statements like “Even though patients were struggling, my role as a nurse didn’t change.” This sense of mission provided a strong motivator and source of fulfillment. Furthermore, the subcategory “Insights gained from experience,” emerged as a significant reward, with nurses recognizing the substantial professional growth they experienced. They viewed their experiences as an opportunity for personal growth, gaining new insights and confidence through their work, as one nurse stated, “It gave me a chance to build confidence.” and another reflected, “Being assigned to the COVID-19 ward changed the way staff felt empowered” demonstrating the positive personal and professional development stemming from these challenging times. Some simply found “It turned out to be an incredible experience.”

The second theme, Formation of New Human Connections, captured the unexpected positive outcomes related to new relationships formed during the pandemic. “Collaboration among staff” was a key subcategory, with nurses reporting a strengthened sense of teamwork and mutual support. Working with colleagues from different departments helped them to form new bonds and a shared sense of purpose in facing the common challenge, nothing “I was able to connect with colleagues from other departments” and “I heard many different perspectives from staff across various wards.” This collaboration extended to “Unity as an organization,” as some nurses perceived a greater sense of organizational cohesion amidst the crisis, feeling that “The wards became more unified” and “We truly came together as one team.” This unity, forged through shared adversity where “Discussions about how to manage the wards brought people together”, provided a crucial source of resilience and support, demonstrating the unexpected positive social outcomes of navigating the pandemic together.

## Discussion

This study revealed that nurses with experience in COVID-19 wards faced a complex interplay of occupational distress and job-related rewards. Consistent with prior research ( Awano et al., 2020), these nurses grappled with fears directly related to COVID-19, such as the fear of infection. This aligns with the broader body of research focusing on the stress and psychological impact faced by healthcare workers during the COVID-19 pandemic ( Arias et al., 2023). The findings of the current study show how nurses working in infectious disease wards confronted unique dilemmas and distress. Three primary themes emerged from the interviews: fear of contagion, interpersonal conflicts, and organizational challenges. Additionally, the pandemic’s impact extended beyond the individual nurse to their families, placing nurses in a difficult position of balancing personal and family safety with their professional obligations ( Catania et al., 2021). In such scenarios, a safe and secure organizational environment is crucial for enabling nurses to fulfill their duties. However, previous reports have indicated that during the COVID-19 pandemic there was a stronger sense of distrust towards organizations and conflicts in interpersonal relationships among nurses ( Soto et al., 2020). This study also identified similar conflicts, with participants describing tensions between departments and communication breakdowns. It is likely that accumulation of such conflicts over time will lead to distress and potentially to burnout. Furthermore, lack of communication and support within organizations might have exacerbated nurses’ stress, forming the root cause of their distress and dilemmas. Organizational support is crucial in maintaining nurses’ mental health ( Labrague, 2021), with managerial presence, information provision, and attentiveness to subordinates’ opinions reducing nurses’ anxiety and facilitating mutual understanding under challenging work conditions ( Skogsberg M. et al., 2022). Together, these insights call attention to the need for sustained, empathetic organizational responses that prioritize both the professional and personal well-being of frontline nurses, particularly in times of healthcare crises.

Importantly, this study also revealed certain positive aspects, which emerged as distinct themes in the qualitative analysis. Despite the challenges faced in Covid-19 wards, participants consistently reported experiencing rewarding aspects to their job rewards, a sense of mission, and the forging of new relationships and connections, despite the challenges faced in COVID-19 wards. For example, several nurses described a renewed sense of purpose when witnessing patient recovery, and many reported strengthened collegial bonds formed through shared adversity. These finding could be related to the joy and rewards healthcare workers derive from helping others and reconnection with a sense of vocation. Reports suggest that contributions to patient care and a sense of accomplishment positively impacted healthcare workers’ mental health during the COVID-19 pandemic ( Yamada et al., 2022; Trumello et al., 2020). These findings imply that working in COVID-19 wards and responding to patients’ needs can cultivate a sense of professionalism and fulfillment. Onodera (2022) noted that in wards temporarily formed with members from different departments, the ability to collaborate is crucial for enhancing caregiving capabilities. Collaboration involves building relationships with team members, identifying and solving workplace problems, understanding the abilities of members from different specialties, and appropriately delegating tasks. The findings of the current study suggests that nurses from various wards working together shared diverse expertise, leading to the formation of staff collaboration and organizational unity. Working while forming new human connections may have also enhanced nurses’ caregiving capabilities. The novel environment of the COVID-19 wards potentially enhanced the professional capabilities of each individual, as they were compelled to utilize their skills to the fullest. A key factor in drawing out these abilities may have been the necessity to form relationships with colleagues they had never worked with before, as evidenced by multiple participants who described the development of new problem-solving approaches within their ad hoc teams.

Nurses, as healthcare professionals, faced the need to respond to the unfamiliar challenge of COVID-19, carrying with them feelings of anxiety and distress. In this context, they not only experienced personal growth triggered by COVID-19 but also expanded their professional connections through their pandemic experiences. The tension between occupational distress and professional fulfillment identified in this study extends previous research by Trumello et al. (2020) and Labrague (2021), suggesting that nursing during crisis situations creates a unique professional working environment where challenges and rewards coexist. This dialectic between struggles and rewards deserves further exploration in future studies, particularly regarding how healthcare institutions might encourage the positive aspects while lessening the negative impacts of crisis response work. Additionally, longitudinal research impacts of how these experiences shape nursing practice beyond the pandemic would examining how these experiences shape nursing practice beyond the pandemic would contribute valuable insights to the field.

### Limitations and future prospects of this study

This study is limited by its focus on nursing professionals from a single facility, rendering the results non-generalizable. To construct a comprehensive theory, further data collection and analysis involving other institutions and a larger sample size are necessary. Additionally, capturing temporal changes was challenging, necessitating ongoing monitoring of the situation.

Based on the insights gained from this research, it is essential to conduct quantitative studies to further deepen our understanding of the various conflicts challenges that nurses face, especially when confronted with a large-scale medical emergency. This understanding would contribute to the development of effective strategies and interventions to support nurses during difficult times. Moreover, a more detailed examination of the types of psychological support that can be offered to nurses is required to effectively address their specific needs and challenges. Nurses, as professionals, faced the necessity to respond to COVID-19, an unknown infectious disease, which brought with it severe feelings of anxiety and distress. Amidst these challenges, the participating nurses in the current study also experienced personal growth and an expansion of connections with others, triggered by COVID-19. This has revealed how they, as professionals, are grappling with conflicting experiences of both distress and fulfillment, and their perspectives on how to perceive and manage this tension moving forward. Further investigations into both the negative and positive aspects brought by the pandemic can help us to understand how to support nurses in their vital caregiving work.

## Conclusion

This study sought to clarify the distress and dilemmas experienced by Japanese nurses working in COVID-19 wards. It revealed that nurses faced significant challenges, including conflicts related to the risk of infection, challenges in fulfilling job roles, organizational conflicts, and difficulties within interpersonal relationships. Despite these challenges, nurses also found positive aspects such as job rewards and opportunities to form new human connections throughout their experiences working on COVID-19 wards. These findings indicate that while nurses bore the distress and burden related to COVID-19, which led to mistrust towards their organizations, they simultaneously discovered new aspects of job satisfaction and the value of engaging with people they had not previously encountered. Our findings illustrate the dilemmas faced by professionals in handling the distress inherent in their roles and balancing that with their sense of professional duty and vocational rewards. The insights gained from our investigation into the experiences of nurses working on COVID-19 wards at the height of the deadly pandemic can help healthcare providers to devise effective strategies and interventions to support nurses in their important work.

## Authors’ contributions

Asako Matsuura contributed to the research design, data collection, analysis and interpretation, and drafting of the manuscript. Shin-ichiro Sasahara was involved in conceptualizing the study, research design, recruiting participants, and contributed to data collection, analysis, and interpretation. Hirokazu Tachikawa contributed to the conception of the study, interpretation of the data. Yoshitaka Kawashima and Sho Takahashi contributed to interpretation. Shotaro Doki, Daisuke Hori, and Tsukasa Takahashi assisted in recruiting participants and contributed to data analysis and interpretation. Masana Ujihara and Keiko Wataya contributed to the analysis and interpretation of research data and critically revised the content of the manuscript. Kei Muroi contributed to the critical revision and English proofreading of the manuscript. All authors discussed the results critically and approved the final version of the manuscript.

## Data Availability

Parts of the data used in this study are available for public access at Matsuura (2024). However, from the perspective of personal information protection, specific details of certain datasets remain confidential. This includes data deemed inappropriate for public release because it contains information that could identify individuals. For detailed inquiries about the use of data in this research, or if you wish to request limited access to the data, please contact the principal investigator directly. Zenodo: Distress and rewards of nurses with experience in COVID-19 wards. https://zenodo.org/doi/10.5281/zenodo.10616718 ( Matsuura, 2024). This project contains the following data:
- Interview Data.docx - interview guide.docx -
Table 1.xlsx -
Table 2.xlsx -
Table 2_code_V3.xlsx -
Table 3. xlsx -
Table 3_code_V3.xlsx Data are available under the terms of the Creative Commons Attribution 4.0 International license (CC-BY 4.0).
