# Supplementary material for: The Dilemmas, Distresses, and Rewards Experienced by Nurses Working in COVID-19 Wards: A Qualitative Study
Source: F1000Res. 2025 May 29;13:559. Originally published 2024 May 31. [Version 3] doi: 10.12688/f1000research.147675.3 (PMC12477463; doi:10.12688/f1000research.147675.3)
Supplement: Peer review history

# Reviewer report: Reviewer response for version 3

**DOI:** 10.5256/f1000research.182415.r388482

**Published:** 2025-09-28

**Authors:** Janice Nesbitt

**Affiliations:** 1 Winnipeg Regional Health Authority, Palliative Care Program, Manitoba, Canada

I thank the authors for reviewing recommendations from the reviewers. This is a much improved manuscript. The flow and organization, along with result reporting in a academic format is so much better. My only feedback is one discrepancy--- there were 12 study participants, but in the results section-- under "Challenges in Fulfilling Job Roles...". it is noted "20 participants". Likely inaccurate. As it is published-- feel must approve. Since already approved it seems

Is the work clearly and accurately presented and does it cite the current literature?

No

If applicable, is the statistical analysis and its interpretation appropriate?

I cannot comment. A qualified statistician is required.

Are all the source data underlying the results available to ensure full reproducibility?

Partly

Is the study design appropriate and is the work technically sound?

Yes

Are the conclusions drawn adequately supported by the results?

No

Are sufficient details of methods and analysis provided to allow replication by others?

Yes

Reviewer Expertise:

palliative care

I confirm that I have read this submission and believe that I have an appropriate level of expertise to confirm that it is of an acceptable scientific standard.

# Reviewer report: Reviewer response for version 2

**DOI:** 10.5256/f1000research.178467.r380407

**Published:** 2025-05-14

**Authors:** Aladeen Alloubani

**Affiliations:** 1 Nursing Research Unit, King Hussein Cancer Center, Amman, Jordan

- **Clarify and organize themes and sub-themes** more distinctly. A visual table or conceptual diagram would help readers follow the narrative more easily.
- **Reduce redundancy** across the abstract, results, and discussion sections. Several points are repeated in similar wording without adding analytical depth.
- **Simplify language** and avoid overuse of jargon (e.g., “elucidate,” “interrelation”) in favor of more accessible terms.
- **Provide clearer linkage between results and discussion.** When discussing positive outcomes or implications, explicitly tie these to the themes and participant quotations.
- **Address inconsistent terminology** regarding participant roles (e.g., head nurse, deputy head nurse) to improve clarity in demographic reporting.
- **Strengthen discussion** by more deeply integrating findings with the literature and offering specific, practical recommendations for policy or institutional support mechanisms.

Is the work clearly and accurately presented and does it cite the current literature?

Partly

If applicable, is the statistical analysis and its interpretation appropriate?

Not applicable

Are all the source data underlying the results available to ensure full reproducibility?

Partly

Is the study design appropriate and is the work technically sound?

Yes

Are the conclusions drawn adequately supported by the results?

Partly

Are sufficient details of methods and analysis provided to allow replication by others?

Yes

Reviewer Expertise:

Nursing, Public Health, Occupational Health, Qualitative Research, Health Policy, Mental Health in Healthcare Workers.

I confirm that I have read this submission and believe that I have an appropriate level of expertise to confirm that it is of an acceptable scientific standard, however I have significant reservations, as outlined above.

# Reviewer report: Reviewer response for version 1

**DOI:** 10.5256/f1000research.161899.r358289

**Published:** 2025-02-04

**Authors:** Janice Nesbitt

**Affiliations:** 1 Winnipeg Regional Health Authority, Palliative Care Program, Manitoba, Canada

Thank you for the opportunity to review this manuscript.

 In general:

- There is a mix of verb tenses. Would suggest reviewing to ensure they are all consistent.
- The word “elucidate” is used several times in the article. Simpler language would be helpful.
- Tables were helpful, but unfortunately are unable to add value to the reading
- There is much repetition of the same words in different sections. I feel like I get a sense of what the study is and what the findings were, but the organization of presentation is not very clear. I am not certain of what the themes and subthemes truly are.
- I am hesitant to hit approved due to the above, but with some reorganization of the writings, I think there can be value here.

In general-the tables are helpful.

 Under Abstract

- Under Results it has the experience of the team from 4 to 21 years. “Four” should likely be spelt out.
- Under background-the word elucidating will cause people to need to look it up. Simple language would be helpful.
- Under the results section-there is reference to three head or deputy head nurses and then three head nurses. This appears confusing. Perhaps there are six head nurses? It is not clear. The table says 3 head nurses and 3 deputy head nurses. Would suggest modifying.

Under Introduction:

- Paragraph two - could delete the definition of moral dilemmas and reference to Jameton. The whole second paragraph does not clearly add value to the article.
- Under the distressed experience by nurses section, there are many extreme words that are not really needed for reporting a research study. For example, paragraph two. The intro line. “ with regards to the first category” is really not needed. Would suggest using more direct and concise language if possible.
- In paragraph two of the same section, you start the paragraph with “ additionally”. But in reality, you are speaking about the same topic as in the prior paragraph.

Under Results

- I am not certain the formatting of the journal that this is submitted to, but the quotations often are not simply embedded in a paragraph. Would leave the discretion of the editors of the journal.
- The results section is not very clear. There is a brief paragraph with a lot of quotes from participants, but that is not well organized.
- There is no clear listing of the derived qualitative themes that are referenced in the discussion and conclusion, making it hard to follow. I am not certain if this is the actual writing or translation lens.
- The subtitle on “job rewards gained from working in COVID-19 wards” is very brief and does not truly speak to the rich results one might expect to see an qualitative study

Under Discussion

- Would suggest to not use the words” our study” but rather “this study”. Keeps the information and reporting more objective.
- In paragraph two, there is mention of positive aspects found in the study, but these were not highlighted in the findings. These seem to be themes—but it is not clear how we got here. There is no clear outline and connection of the themes of the qualitative research project.
- There is not a robust presentation of a discussion. This section may benefit from sharing the finding more robustly in the content of the existing literature.

Ethics and consent (near end)

- This is a repeated section. It is likely not needed here as was noted at the beginning of the article.

Is the work clearly and accurately presented and does it cite the current literature?

No

If applicable, is the statistical analysis and its interpretation appropriate?

I cannot comment. A qualified statistician is required.

Are all the source data underlying the results available to ensure full reproducibility?

Partly

Is the study design appropriate and is the work technically sound?

Yes

Are the conclusions drawn adequately supported by the results?

No

Are sufficient details of methods and analysis provided to allow replication by others?

Yes

Reviewer Expertise:

Compassion Fatigue, COVID- 19 pandemic

I confirm that I have read this submission and believe that I have an appropriate level of expertise to state that I do not consider it to be of an acceptable scientific standard, for reasons outlined above.

# Reviewer report: Reviewer response for version 1

**DOI:** 10.5256/f1000research.161899.r287201

**Published:** 2024-07-08

**Authors:** Gregorius Abanit Asa

**Affiliations:** 1 Research Centre for Public Health, Equity and Human Flourishing (PHEHF), Torrens University, Adelaide, South Australia, Australia

This is a good article. Below is the review for the improvement of the paper. Thank you.

 1.Introduction

 This dual role has placed them in a position of constant striving for professional excellence amid concerns for personal safety and the risk of transmitting the virus to their families, often leading to social isolation both within and outside the hospital… I think this needs citation.

 2. Aim and title *In abstract:* This study aims to explore the psychological burden and dilemmas faced by nurses working in COVID-19 wards, elucidating their professional distress and rewards, and examining their interrelation. *In the end of introduction:* the aim of the current study was to elucidate both the struggles (occupational distress) and satisfactions (sense of achievement) of nurses who worked in COVID-19 wards, as well as their characteristics, and explore how these aspects are interrelated. The title, distress and Rewards of Nurses with Experience in COVID-19 Wards

 The first aim in abstract mentioned about psychological burden and dilemma. Meanwhile, in the end to introduction directly mentioned about occupational distress and no dilemma. The title goes straight to distress and Rewards which seems no dilemma aspect. I would suggest the aim should be the same, so it is clearer for readers.

 3.  Knowledge gap

 I would suggest the authors cite systematic reviews or other reviews on to justify that the study of the topic is lacking instead of citing individual paper.

 4. Data collection

 The study was conducted from January 2022 to March 2023, with interviews carried out from March 2022 to January 2023. This sentence should be clearer. The interview started from March but the study was conducted from January. The authors need to explain why?

 5. Data saturation. Authors need to explain how data saturation was reached

 6. Did the author have pre determined questioned? If so, the authors need to explain to process of developing questions

 7. The authors need to explain the process of developing and formulating the question

 8. The authors need to explain sampling technique

 9. Suggested reference that could be added in the introduction or discussion. [Ref-1]

Is the work clearly and accurately presented and does it cite the current literature?

Yes

If applicable, is the statistical analysis and its interpretation appropriate?

Not applicable

Are all the source data underlying the results available to ensure full reproducibility?

Yes

Is the study design appropriate and is the work technically sound?

Yes

Are the conclusions drawn adequately supported by the results?

Yes

Are sufficient details of methods and analysis provided to allow replication by others?

Partly

Reviewer Expertise:

My area of research is public health.

I confirm that I have read this submission and believe that I have an appropriate level of expertise to confirm that it is of an acceptable scientific standard, however I have significant reservations, as outlined above.

## References

[1] : The impact of working in COVID-19 hospital on indonesian nurses' mental health and wellbeing: a qualitative study. *BMC Nurs* .2022;21(1) : 10.1186/s12912-022-01131-6 345 10.1186/s12912-022-01131-6 36476610 PMC9727997

# Response

**Published:** 2025-05-14

**Authors:** Asako Matsuura

**Affiliations:** University of Tsukuba, Tsukuba, Ibaraki Prefecture, Japan

Dear Reviewer Gregorius Abanit Asa,

 Thank you very much for your thorough review and valuable comments on our manuscript.

 We sincerely appreciate your insightful feedback, which is of great help in improving the quality of our paper.

 We have carefully considered all of your comments and have revised our manuscript accordingly.

 Our revisions are detailed below.

 Asako　Matsuura
